# A Mandible with the Temporomandibular Joint—A New FEM Model Dedicated to Strength and Fatigue Calculations of Bonding Elements Used in Fracture and Defect Surgery

**DOI:** 10.3390/ma14175031

**Published:** 2021-09-02

**Authors:** Jarosław Mańkowski, Jakub Piękoś, Karol Dominiak, Piotr Klukowski, Michał Fotek, Maciej Zawisza, Piotr Żach

**Affiliations:** 1 Institute of Machine Design Fundamentals, Warsaw University of Technology, 00-661 Warsaw, Poland; prklukowski@gmail.com (P.K.); michal.fotek@pw.edu.pl (M.F.); maciej.zawisza@pw.edu.pl (M.Z.); piotr.zach@pw.edu.pl (P.Ż.); 2 MedDev Engineering, 02-522 Warsaw, Poland; j.a.piekos@gmail.com; 3 Department of Head and Neck Cancer, Maria Sklodowska-Curie National Research Institute of Oncology, 02-034 Warsaw, Poland; kr.dominiak@gmail.com

**Keywords:** fatigue strength, strength analysis, durability assessment, FEM model, mandible, temporomandibular joint, bonding plate, fracture

## Abstract

The aim of the study was to develop a new FEM (finite element method) model of a mandible with the temporal joint, which can be used in the numerical verification of the work of bonding elements used in surgical operations of patients with mandibular fractures or defects. Most of such types of numerical models are dedicated to a specific case. The authors engaged themselves in building a model that can be relatively easily adapted to various types of tasks, allowing to assess stiffness, strength and durability of the bonded fragments, taking into account operational loads and fatigue limit that vary in time. The source of data constituting the basis for the construction of the model were DICOM (digital imaging and communications in medicine) files from medical imaging using computed tomography. On their basis, using the 3D Slicer program and algorithms based on the Hounsfield scale, a 3D model was created in the STL (standard triangle language) format. A CAD (computer-aided design) model was created using VRMesh and SolidWorks. An FEM model was built using HyperWorks and Abaqus/CAE. Abaqus solver was used for FEM analyses. A model meeting the adopted assumptions was built. The verification was conducted by analyzing the influence of the simplifications of the temporomandibular joint in the assessment of mandibular strain. The work of an undamaged mandible and the work of the bonded fracture of the mandible were simulated.

## 1. Introduction

Numerical analyses by means of the finite element method are now increasingly used by teams of engineers cooperating with medical doctors in the development of innovative treatment methods and new solutions used in medicine, e.g., individual implants or reconstruction plates. Thanks to this, the initial testing phase takes place completely virtually without exposing the patient, and only thoroughly tested solutions are qualified for the final stage—clinical trials, which offers a lower risk of errors and complications. The integration of technical and medical environments in recent years has significantly contributed to the improvement of the quality of treatment, and its further consolidation and expansion will ensure continuous development of this field.

In the craniofacial region, an element most often subjected to FEM analyses is the mandible, which is the only movable bone of the skull. For this reason, when performing tests and computer simulations, it is necessary not only to be acquainted with biomechanics, but also the anatomical structure of the mandible. Considering the structure of the mandibular bone, one can distinguish compact bone (compact substance) and spongy bone (spongy substance) [1,2,3,4,5].

The mandible is connected to the skull by temporomandibular joints (right and left). They are the only pair of bilateral joints in the human body functioning as one unit that move simultaneously, regardless of the adopted direction. The temporomandibular joint consists of articular surfaces of the head of the mandible (condylar process), temporal bone, articular disc, capsule and articular ligaments.

The temporomandibular joint is classified as a compound joint, and its movement can be divided into three phases: mandibular extension and retraction—translational movements; mandibular abduction and adduction (mouth opening and closing)—a combination of translational movement and rotation; chewing movements—rotational movement (hinge movement) with possible additional lateral translational movement (lateral shift of the mandible). For most of the time in which the mandible works, translational and rotational movements occur simultaneously in temporomandibular joints [6].

**Figure 1:**
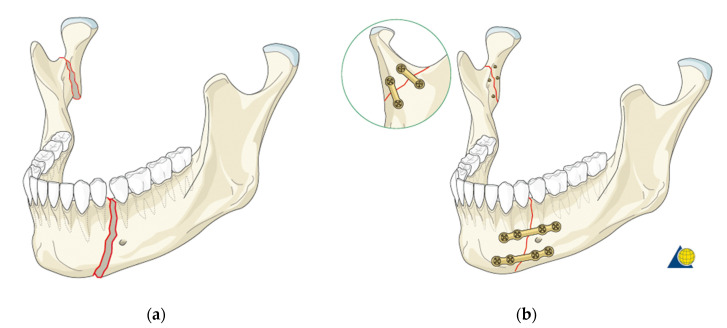
(**a**) Simultaneous fracture of the mandibular body and ramus; (**b**) the effect of bonding both fractures [10].

Due to its location and functions, the mandible is exposed to many mechanical injuries, the most common of which include body or ramus fractures [7,8,9]. The treatment process consists in joining bone fragments and fixing the fracture site with plates (Figure 1). When developing plates, computer simulations and numerical analyses by means of the finite element method are used, which enable a better understanding of the fracture mechanism and the phenomena occurring when the bone is strained. In addition, they allow to quickly check various solutions, e.g., plate shapes, different materials or different connections with the patient’s body [10].

One of the first articles dealing with the analysis of stresses in the human mandible using the finite element method is a publication in the Journal of Cranio-Maxillofacial Surgery from 2000 [11]. Using cadavers, the researchers analyzed the influence of increased load on the values of deformations obtained by carrying out load tests on the mandible using a testing machine. The obtained results showed the linear dependence of deformation on the load, which confirmed the correctness of the application of static analysis using the FEM. The authors of the study carried out numerical analyses of the tested models in order to confirm the adopted assumptions and compare the results with those obtained experimentally. The analysis made use of a simplified voxel-based model of the mandible consisting of cuboids with dimensions of 1.8 mm × 1 mm × 2 mm, while the FEM mesh contained only 7100 elements. The results of the numerical analysis were consistent with the results of the strength tests. However, due to limited possibilities of the software, a complete image of phenomena occurring on the loaded mandible was not obtained. The authors of the publication pointed to great potential for the development of medicine resulting from the use of FEM analyses. They also stated that it is necessary to create more accurate models in order to conduct more detailed studies. Later studies by another team, presented in a 2007 publication [12], confirmed the linear dependence of bone deformation on its load.

Despite simplified models and calculation schemes, the study presented in [11] showed a huge benefit from the implementation of technical solutions in medicine. Software development, both for creating models based on DICOM data and for numerical analyses, has also contributed to the wider use of engineering systems in medical research.

In a publication [13] from 2004, the authors present an analysis of stress distribution on the mandible during an impulse impact. The anatomical model used in the FEM analysis took into account differences in bone structure, dividing it into compact and spongy bone. Individual structures were characterized by different strength properties. The FEM mesh of the model consisted of 30,119 elements, which showed progress compared to the previously discussed article. The contact of the upper surfaces of the condylar process with the acetabulum was defined as the boundary conditions of the analysis, but only a mandible model was used as the computational model. The authors tested two variants of the load by changing the areas of impact. In both cases, the concentration of stresses, apart from the places where the load was applied, occurred on the ramus near the notch of the mandible and in the lower part of the front edge of the mandibular ramus. This is consistent with the actual state of affairs, which is confirmed by the statistics on the incidence of certain types of mandibular fractures [8,9,11].

The authors of [14] from 2008 used a simplified model of the mandible to analyze various methods of the fixation of the fractured mandibular body using several types of plates. The loading scheme took into account only bite forces without muscle forces. In addition, boundary conditions were defined by blocking displacements of the upper surfaces of the condylar process. In addition to determining strain for the plates in individual cases, the displacements of both fixed bone fragments were also determined. The authors of the publication pointed out that the most important variable from the point of view of the stability of the fixation (the criterion of the smallest displacement) was the position of the plate on the bone in relation to the fracture site. The shape of the plate was of less importance in this case.

As a result of the adopted simplifications, the obtained results were highly inaccurate. However, article [14] is an example of an attempt to use the FEM analysis in the development of medical solutions, despite still relatively limited computational capabilities.

More advanced numerical analyses are presented in article [15] from 2009. The researchers used a model of the mandible that included fragments of materials with different properties, namely compact and spongy bone and teeth. The FEM mesh for this model contained 47,525 elements. For the analysis of the fixing plates, a fracture of the mandibular body was simulated, thus dividing the model into two parts. However, the boundary conditions assuming the immobilization of the upper articular surfaces were simplified. In the computational model, the authors omitted the articular disc, stating that neglecting movement in the temporomandibular joint would have no effect on bending the mandible during chewing analyzed in the study.

The conducted analyses concerned the optimization of the dimensions of the fixing plates, and the result of the calculations was the development of a new plate "InterFlex II". The developed plate, while maintaining the required strength, was characterized by smaller dimensions compared to those of the existing solutions. The authors of the publication draw attention to a number of benefits resulting from the use of numerical analyses when developing medical solutions, mentioning, among others, the improvement of the quality of the performed procedures, shortening time and reducing costs.

Similar studies carried out in 2013–2014 [16,17,18] concerned new and existing fixing plates used in the collapse of the condylar process (head of the mandible). The authors of publication [16] present a new shape of the fixing plate used for fractures of the head of the mandible. They compared the plate they developed with one of the solutions available on the market. When defining the conditions of the analysis, the authors drew attention to the rotational movement in the temporomandibular joint, which is significant from the point of view of mandible biomechanics. However, a model of the joint was not used, and only the number of degrees of freedom for the articular surfaces was limited, allowing their movement in only one direction while simultaneously rotating the entire mandible. The assumed loading scheme took into account the system of muscles acting on the mandible, mapping attachment points and force vectors from individual muscles.

As a result, the obtained results showed that the newly developed solution with the use of the plate designed by the authors was characterized by better stability of the fixation, which was indicated by smaller displacements obtained in this case. Thanks to the positive results of numerical analyses, a titanium alloy plate was created and implemented in clinical trials.

In article [17], the researchers attempted to determine stresses on the mandible with various methods of fixation of the fractured condylar process, using models of plates available on the market. For the needs of numerical analyses, a very accurate model of the mandible was created, both in terms of anatomical structure (eight zones consisting of materials with different properties were defined) and biomechanical structure (the loading scheme showing the system of muscles acting on the mandible). The mesh consisted of 1.2 million finite elements. The boundary conditions assumed the possibility of rotation of the mandible around the axis passing through condylar processes, which simulated the hinge movement of the mandible.

By assessing the values of displacements in individual cases, the researchers identified the best solution in terms of the stability of the fixation, i.e., the one in which displacements were the smallest. Moreover, the selected solution was characterized by the lowest strain of the fixing plates and screws used.

Similar studies were also carried out by the authors of publication [18], although they presented a completely different approach to the issue. The publication analyzed three types of plates from a leading manufacturer of this type of solutions. The mandible model took into account differences in bone structure, although only two structures were distinguished—compact and spongy. Another simplification concerned the load, which was defined as a force of 250 N applied to the first molar on one side, thus simulating the load during a unilateral bite. Muscle forces were neglected. Moreover, a simplification of the boundary conditions was applied, reducing them to blocking the displacements of the upper articular surfaces in all directions.

As a result of the adopted simplifications and the stiffening of the system related to the blockage of the displacements of the articular surfaces of the head of the mandible, the obtained results indicated that in each of the analyzed cases, the fixing plates would be destroyed as a result of exceeding the yield point. This calls into question the accuracy of the analyses carried out, given that the plates under examination have been used by medical doctors around the world for years.

Publication [18] is a very good example showing how wrongly adopted boundary conditions influence the obtained results. The results of the conducted research indicate that excessive stiffening of the "structure" may significantly disturb the accuracy of the mapped process.

Summing up, it can be stated that too extensive simplifications of boundary conditions have a negative impact on the results of analyses.

Numerical analyses of this type of structures cause difficulties also due to the need to recreate the anatomical structure and different properties of individual tissues that must be implemented in the computational model. This involves the separation of appropriate fragments with different material parameters. A set of these features determines the degree of mapping of the actual behavior of the object and thus the compliance of the results of numerical analyses with experimental tests.

In connection with the above, the authors of this study formulated the following objectives:

1. Creation of an accurate model of the mandible together with the temporomandibular joint. The main assumption was to create a model that could be used for numerical analyses of various clinical cases, allowing the simulation of the work of fixing elements, assessment of the strength and durability of the fixation, and at the same time, without unnecessary simplifications, reflect real human anatomy. The input data were DICOM images obtained from medical imaging using computed tomography. Obtaining three-dimensional models of individual anatomical structures required the segmentation of tomographic images and then edition of the generated surface objects. Reverse engineering software for free modeling and a CAD program for parametric modeling were used.
2. Taking into account the current achievements and shortcomings of the existing models of the mandible, it was necessary to investigate the effect of simplifications of the temporomandibular joint. Various degrees of simplification of the mandible model were designed, which made it possible to check various configurations of the FEM analysis assumptions and to select the variant that best reflects real conditions. The most important criterion for simplifications was a sufficient reflection of the system biomechanics. This required the creation of a force action scheme based on the actual impact of muscles on the mandible and the introduction of boundary conditions to simulate the appropriate load state. As a result of the performed calculations, maps of stresses in the mandible were obtained, and their analysis allowed to assess the influence of the studied simplifications.
3. Verification of the possibility of modifying the model and applying it in various cases.

## 2. Materials and Methods

### 2.1. Obtaining Anatomical Data

In order to create a model of anatomical structures, it is necessary to provide data in the form of a series of cross-sectional images, synchronized with each other in three planes, most often saved in the DICOM format (digital imaging and communications in medicine—a standard that enables the processing and exchange of images and related medical data). They are obtained during examination using medical imaging techniques, which include, among others, computed tomography (CT, micro-CT, CBCT) or magnetic resonance imaging (MRI, fMRI) [19]. The study made use of data obtained from spiral computed tomography. It is one of the basic methods of medical imaging that uses X-ray properties.

X-ray radiation emitted by the lamp passes through the tested object, being partially absorbed by it, which leads to the weakening of the intensity of the radiation beam to a degree dependent on the thickness and type of the tested material. This causes a change in the radiation intensity, which is described by the relationship [20]:

(1) $$I=I_{0}\cdot X\cdot e^{-\mu g}$$

where:

*I*—radiation intensity after passing through the object,

*I*_0_—initial radiation intensity,

*μ*—linear radiation absorption coefficient characteristic for a given material and a given wavelength of X-rays, 

*g*—thickness of the tested material.

The array of detectors records a change in X-ray intensity and converts it into an electric signal that is digitally processed. The processed signal creates an image in which the brightness of each point corresponds to the absorption capacity of the material. The examined object, after dividing in three planes, is represented by a spatial unit in the form of a cuboid called a “voxel” (the word “voxel” is analogous to the word “pixel”, where vo represents “volume” and el represents “element” [21]). The result of the examination with the use of image processing software [20,22,23,24,25] enables the spatial reconstruction of the geometry of the examined object.

The study was conducted in the Division of Forensic Medicine of the Medical University of Warsaw using a Toshiba Astelion tomograph (Toshiba, Tokyo, Japan). The distance between tomography sections was 0.5 mm.

**Figure 2:**
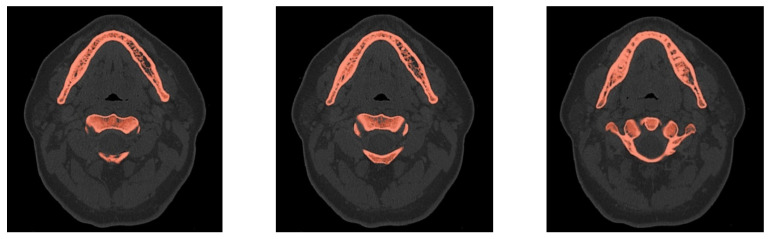
Examples of automated image segmentation for bone structures—3 randomly selected cross sections with a visible bone threshold.

Data series in the DICOM format were digitally segmented [26,27] using the thresholding method [28,29] combined with manually controlled correction of the marked area. The thresholding method consists in singling out an image area containing pixels of a specific color (in this case within the greyscale) by verifying compliance with a specific brightness threshold (value of the threshold parameter). In the case of DICOM images, thresholding causes each section of tomography to be superimposed with a two-dimensional mask covering a given greyscale range. This method enables the identification of areas of different radiological density, using algorithms based on the Hounsfield scale [19,30,31]. Sample results are shown in Figure 2.

### 2.2. Generation of a Three-Dimensional Model of Selected Anatomical Structures

The study made use of the 3D Slicer program (Fedorov A., Beichel R., Kalpathy-Cramer J., Finet J., Fillion-Robin J-C., Pujol S., Bauer C., Jennings D., Fennessy F.M., Sonka M., Buatti J., Aylward S.R., Miller J.V., Pieper S., Kikinis R. 3D Slicer as an Image Computing Platform for the Quantitative Imaging Network. Magnetic Resonance Imaging. 2012 Nov;30(9):1323-41. PMID: 22770690. PMCID: PMC3466397), which is a digital image processing program adapted to handle data in the DICOM format.

**Figure 3:**
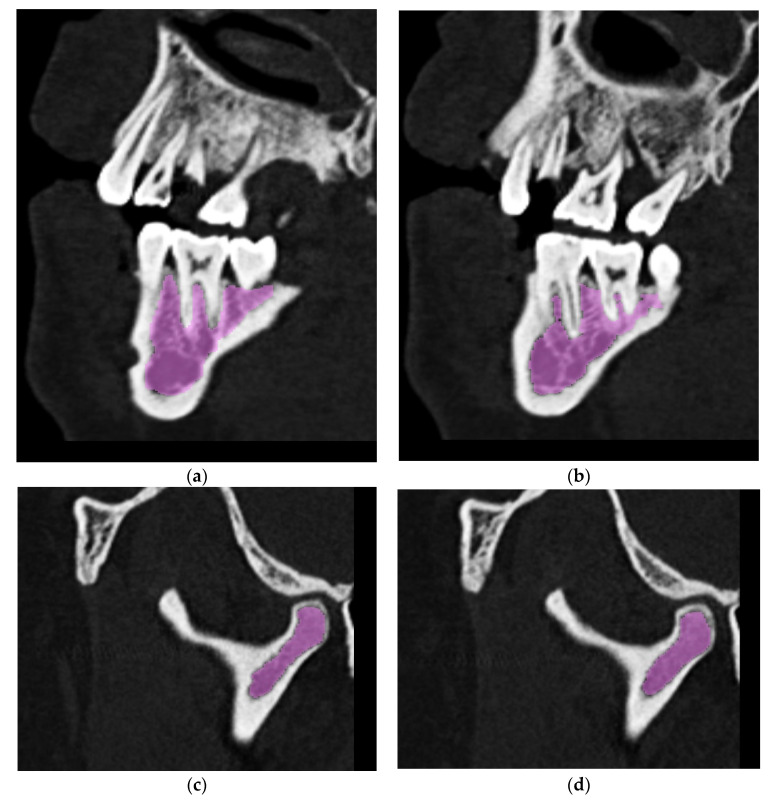
Examples of a hybrid method of singling out the structure of spongy bone in tomographic images: (**a**) exemplary cross section 1—automatic threshold; (**b**) exemplary cross section 1—automatic threshold with "hand" correction; (**c**) exemplary cross section 2—automatic threshold; (**d**) exemplary cross section 2—automatic threshold with "hand" correction.

Due to the high complexity of the geometry and large variation in the radiological density of tissues within the mandible, it is impossible to define such a range of greyscale values as to single out all the required areas in the segmentation process using the thresholding method, especially the spongy bone structure. It was therefore necessary to manually correct the areas marked in the digital image analysis. Thanks to the use of a hybrid method, combining digital processing with manual correction, it was possible to obtain the compliance of the singled-out fragments with the actual anatomical structure of the patient (Figure 3).

After singling out specific areas in the image segmentation process, a voxel model was generated, which was a volumetric composite of two-dimensional masks superimposed on sections in all three planes of tomography. Then, as a result of automatic geometry conversion, the three-dimensional model was saved in the STL format. In this way, a surface model was obtained in the form of the so-called triangle mesh.

Using DICOM data, models representing basic structures within the mandible and the temporomandibular joint were generated. Differences in the structure of the mandibular bone were taken into account, singling out compact and spongy structure, teeth and periodontium. In addition, the articular disc and a fragment of the temporal bone were mapped.

### 2.3. Edition of STL Models

**Figure 4:**
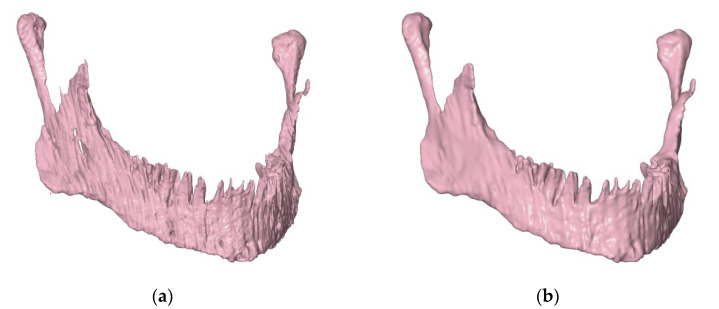
Example effect of "smoothing" the surface of the mandibular spongy structure: (**a**) before smoothing; (**b**) after smoothing.

The demanding and multi-level processing of DICOM data series, including the creation and subsequent joining of two-dimensional masks in all three planes of tomography, may cause difficulties in obtaining a complete image of anatomical structures. This results in errors when generating a three-dimensional model, e.g., surface discontinuities. Models in the STL format were edited in the VRMesh program (V9.4 Reverse, 2017, VirtualGrid, Bellevue, WA, USA) in order to eliminate any imperfections created at earlier stages of work. Additionally, to facilitate the process of creating solid CAD models from the triangle mesh, the models in the STL format were subjected to the “smoothing” operation (Figure 4). In this way, a geometry devoid of the negative layer effect was obtained, resulting from combining tomography sections during the generation of a voxel model. 

### 2.4. Preparation of a Solid CAD Model

**Figure 5:**
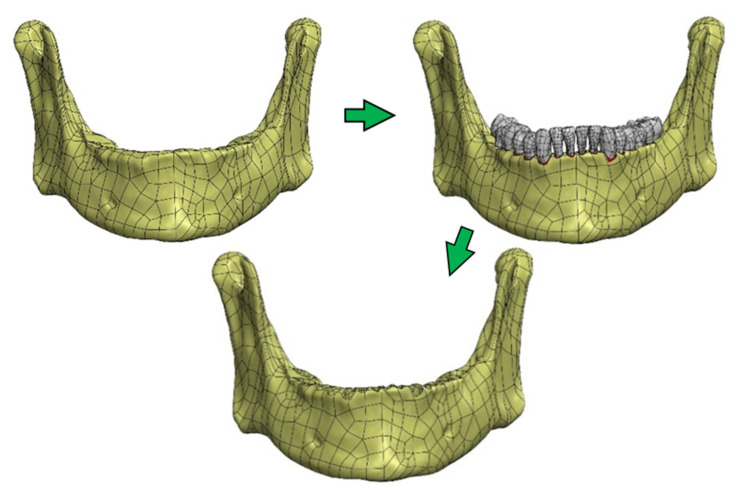
Process of singling out the compact and spongy structure of the mandibular bone.

**Figure 6:**
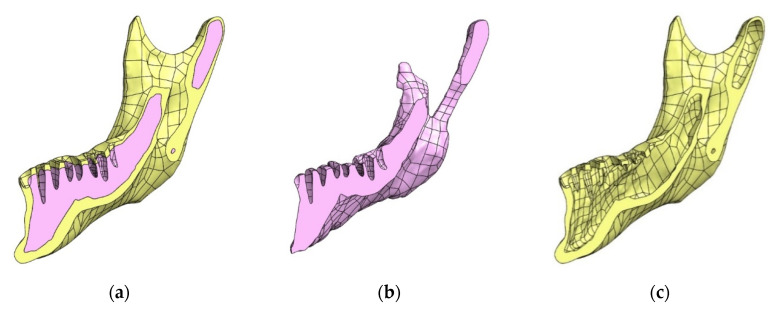
Process of formed the alveolar surfaces: (**a**) the cross sections of the complete mandibular bone geometric model; (**b**) the cross sections of the spongy part the bone structure; (**c**) the cross sections of the compact part the bone structure.

The construction of an FEM model in this case requires a properly prepared solid geometry of the tested object. For this purpose, the SolidWorks program (Solidworks 2018, Dassault Systèmes SolidWorks Corporation, Waltham, MA, USA) was used, in which the geometry of surface models was converted into solid objects. The operations were performed separately for individual structures. In the first step, NURBS (non-uniform rational basis spline) surfaces were generated based on the triangle mesh. Then, the NURBS surface fragments that make up a given model were merged and completed—this allowed to generate solid models. In order to precisely define the contact of individual surfaces and to avoid interferences of models, the final form of objects was created using the Boolean solid subtraction operation [32,33]. In this way, the alveolar surfaces were formed (Figure 5) and compact and spongy parts were singled out in the bone structure (Figure 6).

### 2.5. Preparation of an FEM Model

A finite element mesh was generated using HyperMesh (2014, 2016, Altair Engineering, Inc., Troy, MI, USA) and Abaqus/CAE (2019, 2018, Dassault Systemes Simulia Corp., Johnstone, RI, USA).

### 2.6. Material Data

Based on studies [14,15,16,17,18] and consultations with medical doctors, a detailed analysis of the information on the properties of materials used in numerical models was carried out. The obtained results make it possible to adopt the parameters of material models of bone structures, which are summarized in Table 1.

Data of materials used for fixing plates and screws were verified. In the tested models, screws made of a Ti-6Al-7Nb titanium alloy were used. The material of the fixing plates is Ti-Grade 4. The data included in Table 2, were adopted on the basis of [34] and after modification of the characteristics resulting from [35,36,37,38,39].

### 2.7. Loading Scheme

In studies dealing with the FEM numerical analysis of the human mandible, large discrepancies in the adopted loading scheme can be observed. This is due to a complex system of muscles that move the mandible [3,4].

For many years, work has been underway to develop a loading scheme corresponding to the actual state that is also possible to implement for the needs of analyses [40,41,42,43,44]. For this purpose, in vivo tests, for example, use advanced systems based on electromyography (EMG), which is the measurement of an electric signal related to muscle activation [45,46]. In conjunction with the cross-sectional analysis of the muscle surface, this enables the interaction of individual muscles to be reduced to force vectors. 

The loading scheme was adopted as described in [40] on Figure 3. The model uses a load for one side (right), assuming values for the maximum muscle strength recorded by the authors of the study. The implementation of the loading scheme required a definition of a new coordinate system of the model, identical to the one adopted in [40]. For this purpose, it was necessary to determine the frankfurt plane [47] and the median plane [48]. This was done using the characteristic anthropometric points of the skull and the description of their designation contained in [49]. The process was supervised by medical doctors who confirmed the anatomical correctness of the designated planes.

**Figure 7:**
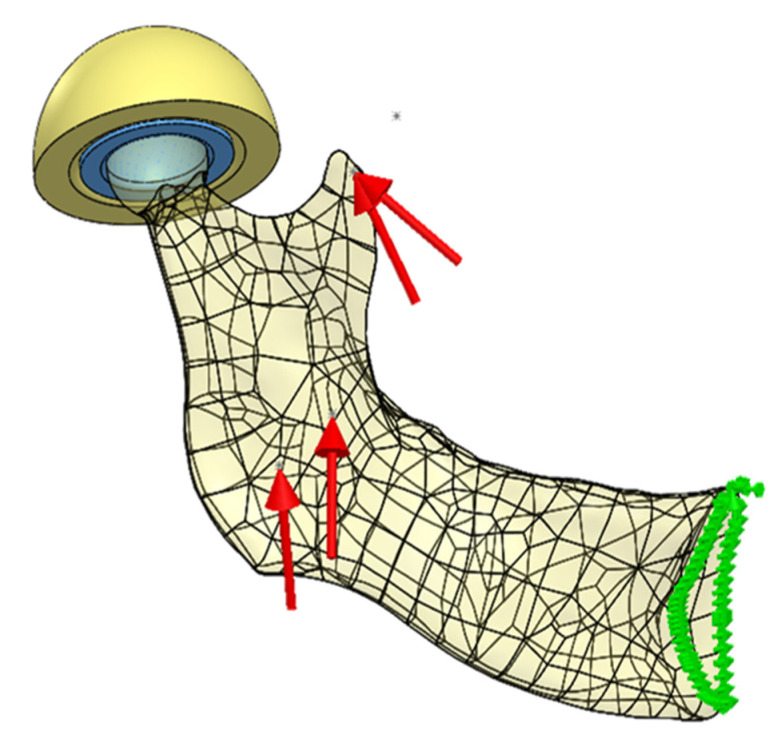
Loads configuration for the analyzed model of the mandible (the red arrows—applied forces, the green arrows—boundary conditions).

The final form of the loads’ configuration adopted for the analyses is shown in Figure 7.

### 2.8. Boundary Conditions

Because of the expected large number of finite elements, half of the mandibular bone was used for test analyses. A simplification was adopted stating that the mandibular bone is a symmetrical structure and boundary conditions resulting from this fact were introduced into the model—a displacement blockade perpendicular to the symmetry plane was introduced for nodes lying in the symmetry plane.

The boundary conditions in the temporomandibular joint were one of the aspects examined during the analysis and changed during subsequent simulations, which was shown in the discussion of the performed analyses.

The remaining blockades of the degrees of freedom were adequate to the task being performed.

### 2.9. FEM Model

Analyses of the tested cases were performed using the Abaqus program. FEM models were generated using various types of solid elements with linear shape functions. In the stress concentration zones and in the contact zones, elements with an average edge length of 1 mm were used. 

The number of finite elements in the prepared test models ranged from 230,389 elements (a simplified model to study the impact of the applied simplifications of the temporomandibular joint) through 949,407 elements (full mandibular model) to 1,058,776 elements (model: fixation work—Martin 3).

## 3. Results

### 3.1. CAD Model

**Figure 8:**
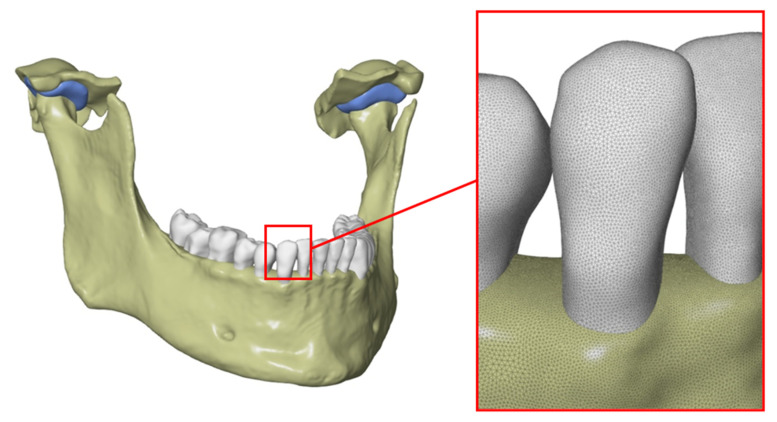
Complete set of STL models of the mandibular bone structures with a close-up showing the density and quality of the triangle mesh.

**Figure 9:**
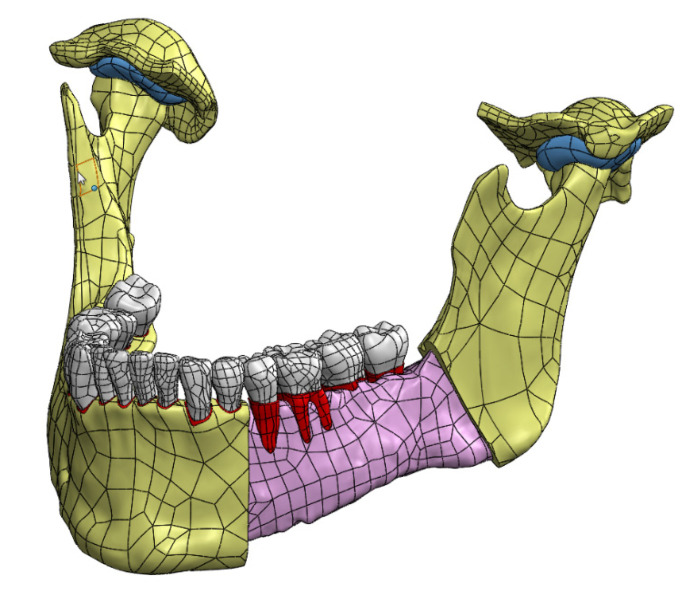
Solid CAD model of the mandible with the temporomandibular joint (with a cut showing internal structures).

The basis for the preparation of a CAD model was the complete set of STL models of bone structures made on the basis of computed tomography (Figure 8). Thanks to it, a very accurate CAD model of the mandible with the temporomandibular joint was created (Figure 9). First of all, the obtained model allowed to generate a finite element mesh for each bone structure separately and to use, for example, a TIE (in Abaqus/CAE - a surface-based tie constraint [50]) bond to ensure cooperation of individual structures. This allowed the model to be optimized in terms of the number of elements. Secondly, due to common surfaces separating individual bone structures, the model allowed to generate a mesh based on common nodes.

**Figure 10:**
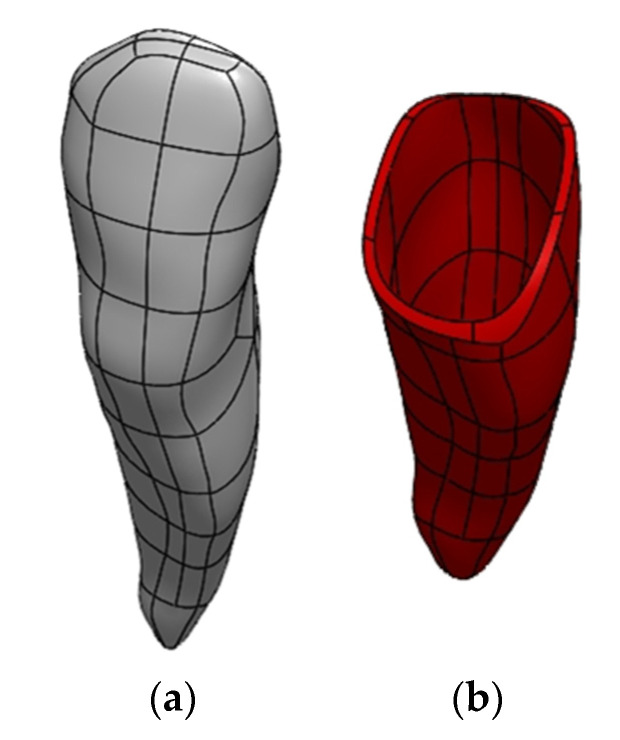
Example of a modeled periodontium for one of the teeth: (**a**) the exemplary geometric model of one tooth; (**b**) the simplified model of periodontium.

Publications [51,52,53] indicate that the periodontium is also an important structure in the mandible model from the point of view of strength analyses. It is the tissue between tooth roots and alveolar processes. The generation of periodontal models was a problem due to small thickness and inability to be accurately depicted. It was decided to generate them as a 0.3 mm thick layer around tooth roots (Figure 10). 

### 3.2. Possibilities of Simplifying the Model

**Figure 11:**
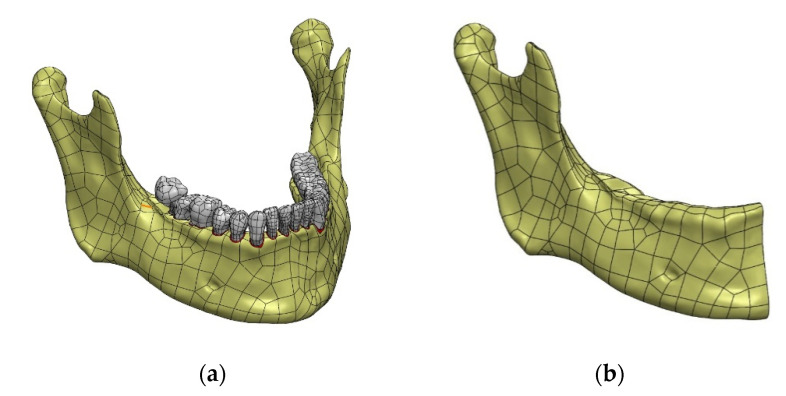
Model simplification and condylar head modification; (**a**) circumscribing a sphere on the bone surface, (**b**) final form of the simplified model.

The prepared model, due to precisely generated surfaces separating individual bone structures, can be easily modified and adapted to any type of analysis. For example, for the purpose of examining the impact of the simplification of the temporomandibular joint on the assessment of the mandibular strain, it was decided to significantly simplify the CAD model by taking into account the numerical analysis of bones without dividing them into individual structures (compact and spongy bone) and omitting tooth models with the periodontium. The quasi-symmetry in relation to the median plane was also used, which is an imaginary symmetry plane (Figure 11).

### 3.3. Examination of the Influence of the Simplification of the Temporomandibular Joint on the Correctness of the Assessment of Mandibular Strain 

#### 3.3.1. Model

**Figure 12:**
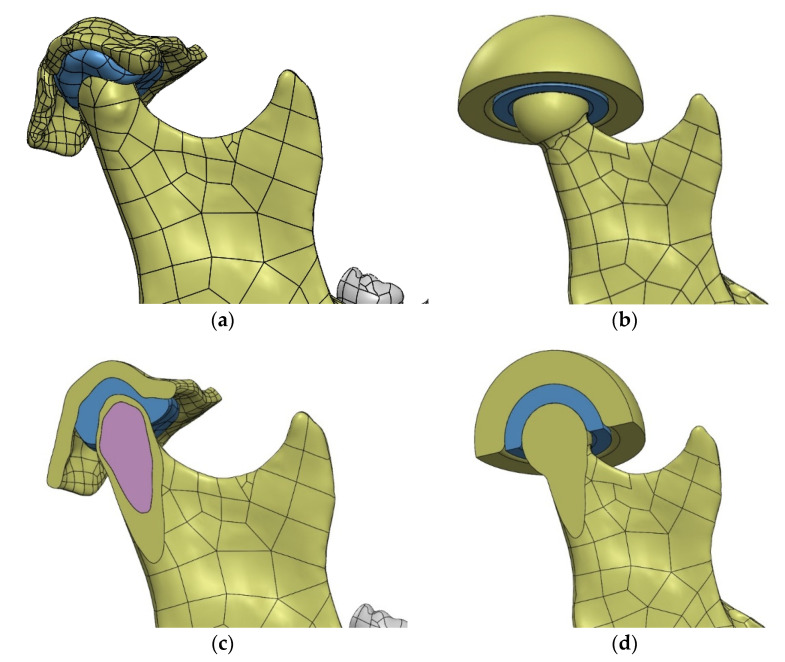
Simplification of the geometry of the temporomandibular joint introduced for the purposes of preliminary FEM analyses in the lateral and cross-sectional view: (**a**) actual model based on tomography; (**b**) simplified model; (**c**) the temporomandibular joint cross section of actual model based on tomography; (**d**) the temporomandibular joint cross section simplified model.

When starting the study, it was decided to further simplify the geometry of the temporomandibular joint. The original geometry was replaced with a spherical joint (Figure 12). The modification of the condylar head was carried out taking into account the anatomical structure, circumscribing a sphere on the bone surface. The adopted simplifications allowed to reproduce the movement of the mandible during chewing, in the initial phase of teeth clenching (hinge movement with possible lateral adduction of the mandible). The analysis excluded the possibility of anterior-posterior mandibular movement, the impact of which, after consultation with medical doctors, was assessed as insignificant in the examined case.

**Figure 13:**
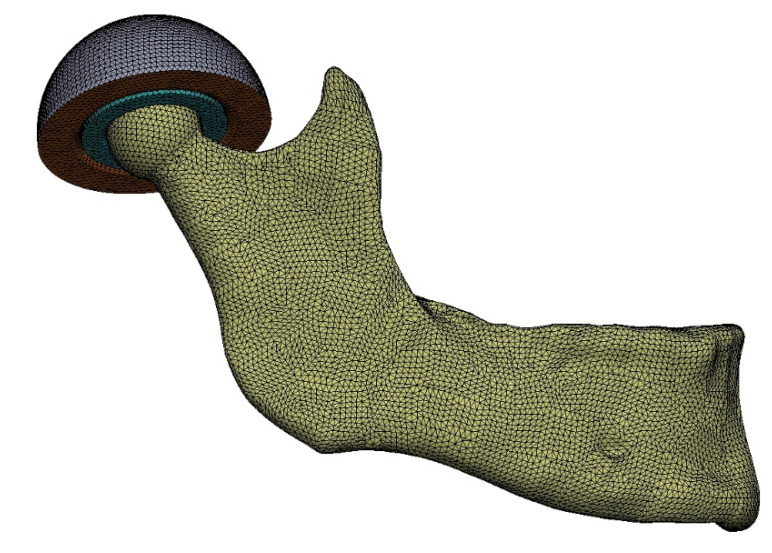
Model of the mandible with the temporomandibular joint with a generated FEM mesh.

In the FEM model (Figure 13), the mandibular bone and the simplified fragment of the temporal bone were given the properties of compact bone material. Simplified articular disc—material properties in accordance with the name.

#### 3.3.2. Examined Cases of Simplifications

Variant 1. Omission of the temporomandibular joint, blocked displacement of the nodes lying on the outer surface of the head of the temporomandibular joint.

Variant 2. Blocked displacement of the nodes lying on the outer surface of the articular disc, non-deformable articular disc, defined contact between the joint head and the disc. 

Variant 3. Blocked displacement of the nodes lying on the outer surface of the articular disc, deformable articular disc, defined contact between the joint head and the disc.

Variant 4. Blocked displacement of the nodes lying on the outer surface of the temporal bone, deformable articular disc, defined contact between the joint head and the disc and between the disc and the temporal bone.

#### 3.3.3. Results of the Experiment

**Figure 14:**
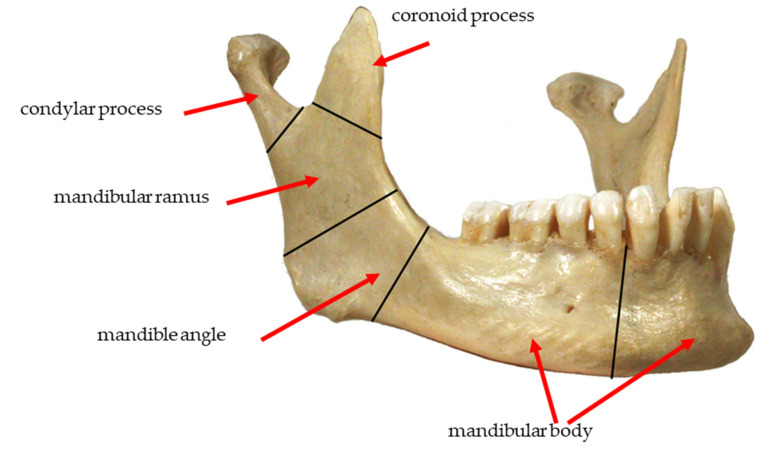
Division of the mandible into typical areas of mandibular fractures [15].

**Figure 15:**
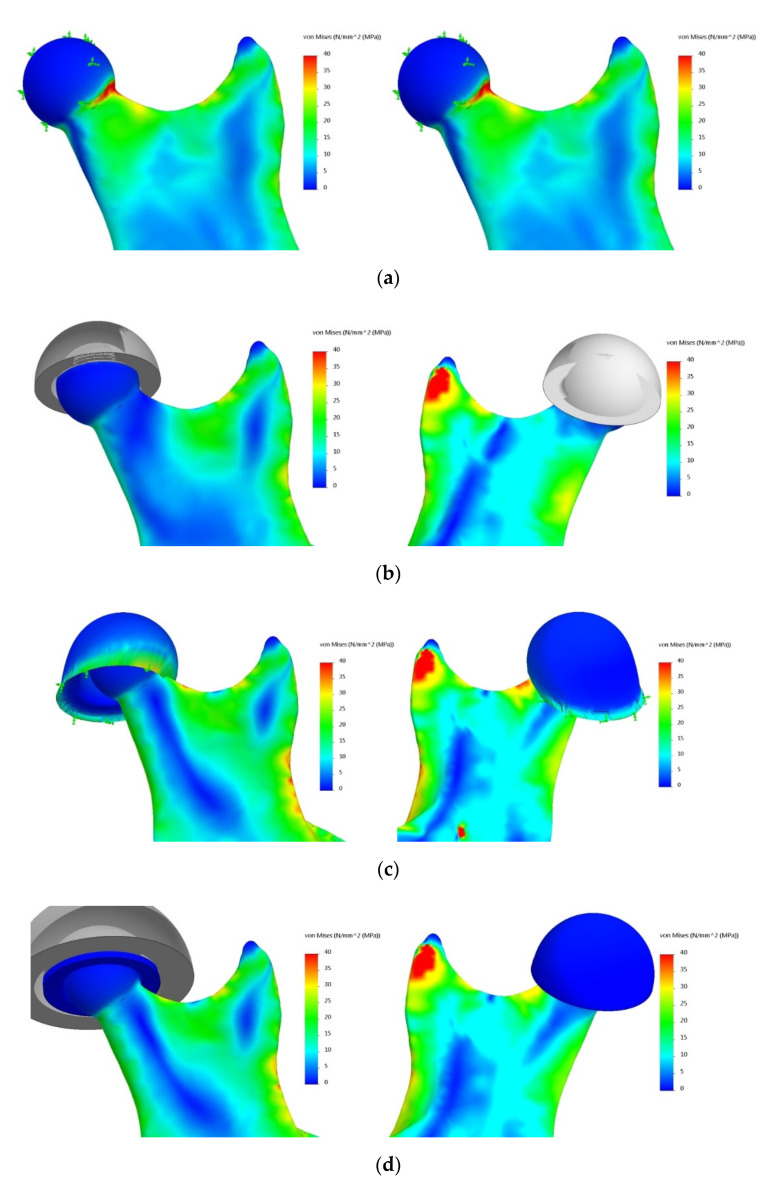
Von Mises stresses—the area of the condylar process (head of the mandible) and the coronoid process: (**a**) Variant 1, (**b**) Variant 2, (**c**) Variant 3, (**d**) Variant 4.

The obtained results were related to the zone according to the classification of the types of mandibular fractures (Figure 14). The von Mises stress distribution for selected sample areas of the condylar process (head of the mandible) and the coronoid process is shown in Figure 15.

## 4. Discussion

The evaluation of the obtained results was made by comparing stress distribution in individual cases in relation to the reference model, which was adopted as a model taking into account the complete structure of the temporomandibular joint. It was carried out in groups divided into areas according to the general classification of mandibular fracture types. For all the tested variants, stress distribution showed characteristic stress concentrations in the vicinity of the places where the load was applied. This was due to the point nature of the action of forces simulating the work of muscles. A significant accumulation of stresses also occurred at a point on the plane of division (symmetry) of the mandible, for which displacements in all directions were blocked.

Using the simplified model, an analysis of the influence of the simplification of the temporomandibular joint and the defined contact conditions between specific structures was carried out in the analysis of mandibular strain. A series of numerical analyses with the use of the FEM were carried out. Their aim was to determine stress distribution on the mandible.

Analysis of the results: After considering the results of the numerical analyses, aimed at determining the impact of simplifications within the temporomandibular joint on stress distribution on the mandible, the following conclusions can be drawn:

- The most simplified model of the mandible with the blocked displacement of nodes on the articular surface of the head of the mandible (Variant 1) should not be used in strength calculations due to large discrepancies between the obtained results and the reference model (Variant 4). The differences can be as high as 216%, as in the case of stresses in the area of the condylar process.
- Simplification of the temporomandibular joint to the model of a non-deformable disc (Variant 2) does not provide a sufficiently accurate representation of the state of mandibular strain, despite recreating the kinematics of the examined bone (hinge movement with possible lateral adductions).
- Modelling the temporomandibular joint as a joint (contact task) has a significant impact on the obtained stress distribution on the mandible (Variants 3 and 4).
- For the analysis of mandibular strain, it is not necessary to build an exact (Variant 4) model of the temporomandibular joint. The reduction of the system to a deformable articular disc provides a sufficiently good representation of stress distribution.
- Simplification of the temporomandibular joint to a deformable articular disc (Variant 3) does not ensure an appropriate state of displacement, which may affect the results of the analysis of fixation stability based on the study of displacement of broken bone fragments.

In addition to the application of the constructed model to assess the simplification of the temporomandibular joint, an analysis of an undamaged mandible work was carried out, as well as a simulation of the work of the mandibular ramus fixation at the level of the condylar process. In both cases, the Martin 3 plate was used, confirming the assumptions.

## 5. Conclusions

The obtained results confirm the thesis that it is necessary to create a new, universal model of a mandible with the temporomandibular joint, which can be used to analyze various cases of surgical treatment of fractures, defects and mandibular corrections. The results of the actual tests and the analysis of the available literature [54,55] allow to state that it is necessary to take into account aspects such as: variable working conditions, changing joint stiffness depending on the load and recovery time and mechanical properties of bone structures dependent on the age and condition of the patient. 

The conducted numerical tests allow to conclude that the developed model can be adapted to various types of tasks. The functional properties of the model make it possible to assess the stiffness, strength and durability of the parts to be fixed. They allow to take into account operational loads varying in time and test the fatigue limit of the mandible with the temporomandibular joint. The results of further research and numerical tests will constitute the content of next articles.

## Figures and Tables

**Table 1:** Material data of bone structures.

Structure	Young’s Modulus (MPa)	Poisson’s Ratio	Re (Compressive Strength) (MPa)
Compact bone	14,700	0.3	159
Dentin (tooth)	14,700	0.3	-
Spongy bone	490	0.3	1.86
Articular disc	6.1	0.49 (0.46 in the analyses due to convergence)	-

**Table 2:** Material data of fixing plates and screws.

Parameter	Ti-6Al-7Nb	Ti-Grade-4
Tensile Strength MPa (ksi) sheet	1000 (145)	552
0.2% Proof Stress MPa (ksi)	900 (130)	483
Elongation over 5D%	12	15
Reduction in Area %	35	30
Elastic Modulus GPa (Msi)	105 (15)	104

## Data Availability

Data is contained within the article.
